# Quantification and Multidrug Resistance Profiles of Vancomycin-Resistant Enterococci Isolated from Two Wastewater Treatment Plants in the Same Municipality

**DOI:** 10.3390/microorganisms7120626

**Published:** 2019-11-29

**Authors:** Haley Sanderson, Rodrigo Ortega-Polo, Kevin McDermott, Geoffrey Hall, Rahat Zaheer, R. Stephen Brown, Anna Majury, Tim A. McAllister, Steven N. Liss

**Affiliations:** 1 School of Environmental Studies, Queen’s University, Kingston, ON K7L 3N6, Canada; 13hs39@queensu.ca (H.S.); gh26@queensu.ca (G.H.); stephen.brown@chem.queensu.ca (R.S.B.); anna.majury@oahpp.ca (A.M.); 2 Agriculture and Agri-Food Canada, Lethbridge Research and Development Center, Lethbridge, AB T1J 4B1, Canada; rodrigo.ortegapolo@canada.ca (R.O.-P.); rahat.zaheer@canada.ca (R.Z.); 3 Department of Biological Sciences, University of Lethbridge, Lethbridge, AB T1K 6T5, Canada; 4 Public Health Ontario, Kingston, ON K7L 3K3, Canada; Kevin.McDermott@oahpp.ca; 5 Department of Civil Engineering, Queen’s University, Kingston, ON K7L 3N6, Canada; 6 Department of Chemistry, Queen’s University, Kingston, ON K7L 3N6, Canada; 7 Department of Biology, Ryerson University, Toronto, ON M5B 2K3, Canada

**Keywords:** vancomycin resistant enterococci, antimicrobial resistance, municipal wastewater, sewage

## Abstract

Wastewater treatment plants (WWTPs) are points of control for the environmental dissemination of antimicrobial resistant bacteria. Vancomycin-resistant enterococci (VRE) were used as indicators of antimicrobial resistance (AMR) in two WWTPs (biologically aerated filter (BAF) and conventional activated sludge (CAS)) in the same municipality. The removal and abundance of enterococci and VRE as well as the species and antimicrobial resistance profiles of VRE were assessed. Enterococci and VRE from the primary and final effluents were enumerated. Results were assessed from an ecological context. VRE was not selected for by either WWTP but the BAF system outperformed the CAS system for the removal of enterococci/VRE. *Enterococcus faecalis* (*n* = 151), *E. faecium* (*n* = 94) and *E. casseliflavus*/*E. gallinarum* (*n* = 59) were the dominant VRE species isolated. A decrease in levofloxacin resistance in enterococci was observed in the BAF WWTP. An increase in nitrofurantoin resistant (*p* < 0.001) and a decrease in quinupristin/dalfopristin (*p* = 0.003) and streptomycin (*p* = 0.022) resistant enterococci were observed in the CAS WWTP, corresponding to a shift of VRE from *E. faecalis* to *E. faecium*. Wastewater treatment processes can be managed to limit the dissemination of antimicrobial resistance determinants into the surrounding environment.

## 1. Introduction

Antimicrobial resistance (AMR) is a growing public health concern. With the decline in the development of new antimicrobials, and the on-going emergence of AMR, limiting the spread of resistance determinants is imperative. Most of the attention for curtailing the spread of AMR has focused on healthcare, veterinary and/or agricultural settings. The majority of antimicrobial agents used in humans occur in the community [1]. Antimicrobial agents are not always fully metabolized by the host, and can be excreted via urine and feces into sewage systems; where in developed nations they eventually reach wastewater treatment plants (WWTPs). While concentrations of antimicrobials in wastewater are significantly lower than therapeutic doses, the microbial ecology of the wastewater remains impacted and selection for antimicrobial resistant bacteria occurs [2]. Wastewater treatment plants (WWTPs) can remove antimicrobials by sorption and/or degradation. However, the activity and fate of antimicrobials in the final effluent depends on their chemical properties among other factors [3]. WWTPs also provide an environment favorable for horizontal gene transfer as they contain high nutrient concentrations and densities of both pathogenic and environmental bacteria subject to periods of stress and recovery during treatment [4]. WWTPs release effluent, containing AMR microorganisms, into both aquatic and terrestrial environments where they have the potential to contact humans [5,6]. Understanding the importance of the continuous input of resistant bacteria and antimicrobial agents into the environment and the impact of the type of WWTP on AMR bacteria would bolster the information needed to assess the role of WWTPs in the dissemination of AMR.

Enterococcal communities in wastewater reflect the commensal microorganisms of the human population within a particular area [7] and provide insight into the impact of human activity on the microbial ecology of water. The carriage of AMR by commensal bacteria, like *Enterococcus* spp. and *Escherichia coli*, has been proposed to be an indicator of the burden of AMR in a population [8], and a means of formulating public health policy [9]. *Enterococcus* spp. are part of the natural intestinal flora of humans and animals, are widely distributed in a variety of environments and are already used as an indicator of fecal contamination, along with *E. coli*, in food products and water [10].

The high prevalence of AMR in enterococci suggests that they are an important agent for the dissemination of resistance at both the intra- and interspecies levels [11]. Vancomycin-resistant *Enterococcus faecalis* and *Enterococcus faecium* are often multidrug resistant, which is problematic given that vancomycin is a drug of last resort for treating infections caused by Gram positive bacteria [12]. While the rates of morbidity and mortality associated with VRE infection are a concern, the spread of vancomycin resistance determinants to other pathogenic organisms, like methicillin-resistant *Staphylococcus aureus* also poses a risk [13]. Acquired vancomycin resistance is common in *E. faecium* and *E. faecalis* and these species are responsible for most nosocomial VRE infections [14,15]. Given that VRE are common in wastewater, they may serve as potential indicators of AMR risk within WWTPs.

The aim of this study was to evaluate the effects of wastewater treatment on the presence and fate of vancomycin-resistant enterococci (VRE) among enterococci of wastewater origin. Two WWTPs with different treatment processes in the same municipality were sampled over two years, and the removal of enterococci and VRE, the operational conditions, performance and service areas of the WWTPs assessed. Additionally, genes related to gene transfer (class I integrons (IntI-1)) and vancomycin resistance (vanA) were enumerated using qPCR. Other aims of this study included determining the prevalence of different VRE species and the antimicrobial resistance profiles of VRE in wastewater before and after each of the two wastewater treatment processes.

## 2. Materials and Methods

### 2.1. Wastewater Treatment Plant Parameters, VRE Surveillance and Antimicrobial Use Information

Two full-scale WWTPs, consisting of a conventional activated sludge (CAS) and a biological aerated filter (BAF) system were sampled biweekly from September 2014 to September 2016. Both WWTPs chlorinated the final effluent and were within the municipality of Kingston, Ontario, Canada with a population of ≈117,660 inhabitants at the time of sampling. While both WWTPs were located in the same municipality, they processed different waste streams. The flow rates for the CAS and BAF systems were 38,800 m^3^/day and 53,076 m^3^/day, respectively. The average solid retention time for the CAS system was 8–12 days as opposed to 2–6 days for the BAF system.

Surveillance of VRE infection rates in hospitals is not mandatory, but Kingston General Hospital does report infections per 1000 patient days as part of its Patient Safety Indicator Reports. During the sampling period, the average was 0.025 cases per 1000 patient days [16]. This is below the Canada-wide rate of 0.045 cases per 1000 patient days for 2014 [17]. The majority of antimicrobials used (93%) are dispensed in the community with a total of 17.8 daily defined doses per 1000 inhabitants per day (DIDs) compared to 1.4 DIDs purchased by hospitals each year [17]. In 2014, the total amount of antimicrobials dispensed through Ontario community pharmacies was 6–6.99 defined daily doses (DDDs) per inhabitant and 4–4.99 DDDs per inhabitant by hospitals [17]. Use of vancomycin in hospitals participating in the Canadian Nosocomial Infection Surveillance Program (CNISP) was 40–50 DDD per 1000 patient days [17]. The use of vancomycin in Kingston area hospitals was not available.

Information on antimicrobial sales collected from community pharmacies in the Kingston and Amherstview (ON, Canada) region was purchased from a multinational medical information and clinical research company, IQVIA (Kirkland, QC, Canada). Information on antimicrobial use in hospitals was unavailable. Antimicrobial sales were defined as the number of prescriptions and the number of units sold by pharmacies for individual antimicrobials for each month during the sampling period.

### 2.2. Land Use

Land use was evaluated using geospatial data from 2013, retrieved from the City of Kingston via the Queen’s University library. ArcMap (version 10.5, ESRI, Toronto, ON, Canada) was used to generate a map containing only the land use within the service areas of the two WWTPs. The total area and percentage of area covered by each land designation were extracted from the ArcMap database. This geospatial information included the amount of land cover designated to a variety of activities including commercial, industrial, residential and institutional uses. Land cover was defined as the amount of surface area used for a particular purpose and did not necessarily reflect the population density of the area. Data on population density within the service areas was unavailable. It should be noted that both WWTPs also received septic waste from rural areas surrounding the municipality, but these data were not incorporated in the land use analysis. The dataset included the land cover associated with a limited inventory of landmarks and major institutions, such as hospitals, universities and military bases.

**Figure 1:**
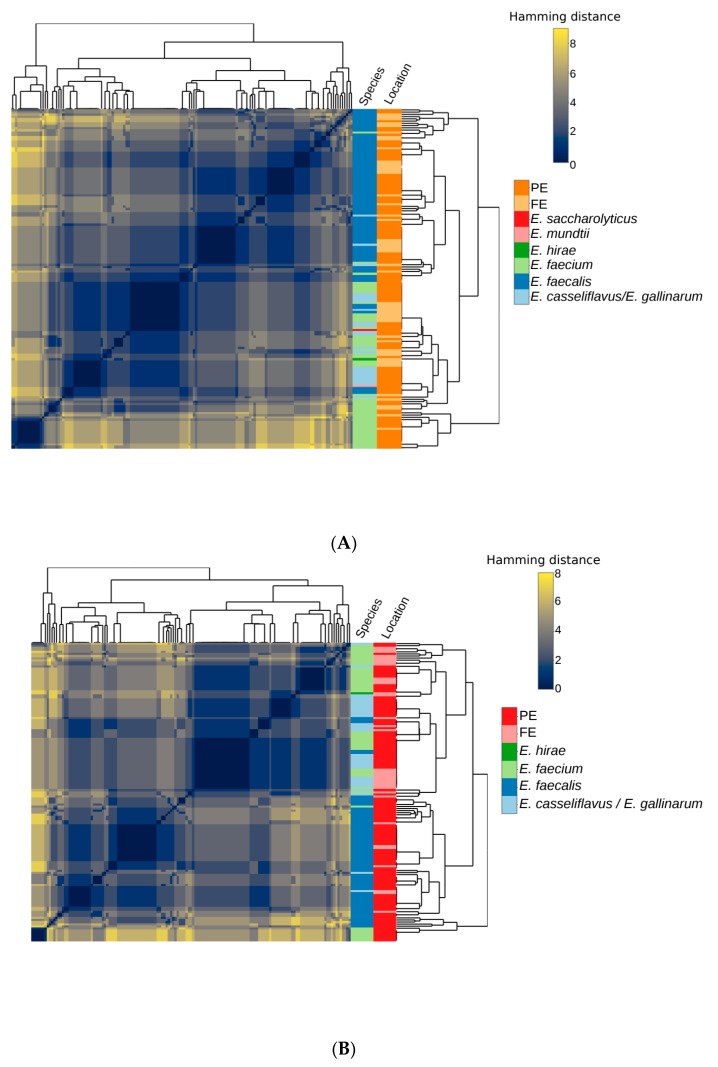
The Hamming distances between antimicrobial resistance (AMR) profiles of isolates from the (**A**) biological aerated filter (BAF) system and (**B**) conventional activated sludge (CAS) system. Distance between the profiles is depicted as a gradient of yellow (large distance) and blue (short distance). The source of the isolates is depicted as primary (red/orange) or final effluent (pink/yellow) in the bar to the left of the heatmap. The species are depicted as *E. hirae* (dark green), *E. faecium* (light green), *E. faecalis* (dark blue) and *E. casseliflavus*/*E. gallinarum* (light blue) in the bar to the left of the heatmap. The tree on the top and left of the heatmap depicts the phylogenetic relationship among isolates and the heatmap depicts the one to one comparison of isolates.

The BAF system serviced a larger area (90,127,296.69 m^2^) than the CAS system (69,378,913.60 m^2^; Figure S1, Table S1). A large part of the area serviced by BAF (49.4%) and CAS (32.8%) WWTPs were protected parkland and nature reserves (Figure 1, Table 1). The BAF system serviced more area designated as institutional use (12.1%) than the CAS system (4.0%) and included hospitals and health units, universities and colleges, as well as a military base and the downtown core. In the Kingston downtown core, municipal sewers and storm water drains were often combined [18]. Whereas, the CAS system serviced more areas designated to commercial, industrial and residential use than the BAF system.

### 2.3. Chemical and Environmental Information

Routine chemical and microbiological testing results for both WWTPs were provided by Utilities Kingston. Routine testing is completed biweekly for alkalinity, pH, biological oxygen demand (BOD), total suspended solids (TSS), phosphorus, total Kjeldahl nitrogen (TKN), ammonia, nitrite and nitrate content. Heavy metals were measured quarterly in the second year of sampling (March–September 2016) and included arsenic, cadmium, chromium, cobalt, copper, lead, mercury, molybdenum, nickel, potassium, selenium and zinc in the primary and final effluent of both WWTPs. Environmental information collected included both air temperature at the time of sampling and precipitation on the day of collection, as retrieved from Environment Canada archives.

### 2.4. Sample Collection, Enumeration and Isolation of Enterococcus

Three types of samples were collected, primary effluent (PE), final effluent (FE) and biomass. The PE was untreated wastewater that had been separated from bulk solids in the primary clarifier before biological treatment and represented the enterococci population before biological treatment. FE was treated wastewater that was sampled prior to its release into Lake Ontario, representing the enterococci population after biological treatment and disinfection. Biomass samples consisted of the biosolids from the biological treatment chamber of each WWTP. Biosolids from the WWTPs in Kingston were applied to agricultural land, but the intermittent availability of biosolids limited their inclusion in the analyses so only a few isolates from this source were included.

Samples of PE (500 mL) and FE (500 mL) were collected in sterile Nalgene wide-mouth natural HDPE bottles (Thermo Fisher Scientific, Toronto, ON, Canada), kept at 4 °C during transport and processed within 8 h of collection. PE samples were filtered through Whatman 1 followed by Whatman 40 filter paper (Sigma-Aldrich, Oakville, ON, Canada) to remove bulk solids. The supernatant was then diluted 10-fold and triplicate diluted samples (0.5 mL) were filtered through a 0.45 µm mixed cellulose filter membrane (Pall Corporation, Mississauga, ON, Canada). The membrane was transferred onto Slanetz and Bartley media (Oxoid Canada, Nepean, ON, Canada). For the FE, 100 mL from the BAF and 10 mL from the CAS system were filtered through a 0.45 µm mixed cellulose filter membrane, plated onto the same media as PE and incubated at 37 °C for 48 h. The difference in volume of FEs between WWTPs was necessary to obtain plates with between 30–300 colonies. Colonies that appeared red, maroon and/or pink were considered presumptive enterococci.

From each plate, 10 colonies were selected and grown on tryptic soy agar (TSA; Sigma Aldrich, Oakville, ON, Canada) with esculin (1 g/L; Sigma Aldrich) and ferric ammonium citrate (FAC; 0.7 g/L; Ward’s Science, New York, NY, USA). Colonies exhibiting a black halo were confirmed as *Enterococcus* spp. by conventional PCR using primers (5′-GAGAAATTCCAAACGAACTTG-3′) and (5′-CAGTGCTCTACCTCCATCATT-3′) for 23S rRNA as described by EPA Method 120013 [19]. Approximately, 1200 confirmed enterococcal isolates were stored for analysis in Todd-Hewitt Broth (THB; Sigma Aldrich) with 7.5% dimethyl sulfoxide (DMSO; Sigma Aldrich). After colonies were chosen from the Slanetz and Bartley agar, the filter was pressed onto TSA with esculin (1 g/L), FAC (0.7 g/L) and vancomycin (4 mg/L or 32 mg/L; Bio Basic, Markham ON) and incubated at 37 °C for 18 h with initial enumeration at 8 h. The number of colonies that produced black halos on this media were counted and considered to be presumptive VRE.

### 2.5. Quantitative TaqMan Real-Time PCR

At each sampling between March 2015 and September 2016, 100 mL of both primary and FE were filtered directly onto a 0.45 µm mixed cellulose filter membrane in triplicate. DNA was extracted from the filters using a PowerWater© DNA isolation kit (Mo Bio Laboratories Inc., Carlsbad, CA, USA) and stored at –20 °C.

Four gene targets were quantified as part of this study. One was a marker for total bacteria, which amplified and quantified a conserved region of the 16S rRNA gene. Enterococci were quantified using the 23S rRNA genes as described by EPA [19]. A vancomycin resistance gene, vanA, was also quantified as an indicator of vancomycin-resistance as it is one of the most common vancomycin resistance genes in wastewater [20]. The presence of class I integrons was assessed using IntI-1 as per a previous study [21].

The primers, probes, standards and references for the assays are reported in Table S2. The specificity of the previously published assays was confirmed using the BLAST program of NCBI (http://www.ncbi.nlm.nih.gov/BLAST/). Reactions used primer concentrations of 400 nM and probe concentrations of 200 nM. Reactions were prepared in a final volume of 25 µL, using MicroAmp Optical 96-well reaction plates (Thermo Fisher Scientific), and contained the following components: 12.5 µL Taqman Environmental Master Mix 2.0 (Life Technologies, Carlsbad, CA, USA), 5 µL of diluted DNA extract, 1 µL of forward and reverse primers and the probe with Black Hole Quenchers (BHQ), 4.5 µL of UltraPure distilled water (Thermo Fisher Scientific) and a variety of reporters (Table S2). Amplification reactions were run using a ViiA^tm^ 7 Real-time PCR system and the data were analyzed using Applied Biosystems ViiA^tm^ 7 Software v1.1 (Applied Biosystems, Foster City, CA, USA). For each reaction, there was an initial cycle of 50 °C for 2 min followed by one cycle of 95 °C for 10 min then 40 cycles of 95 °C for 15 s, followed by an extension step at 60 °C for 1 min.

A standard curve was generated for each assay, using dilutions of genomic DNA or a synthetic plasmid-borne DNA sequence manufactured by Biosearch Technologies (Petaluma, CA, USA) for Class 1 integrons, IntI-1. The 16S rRNA universal bacterial assay used a standard created by pooling genomic DNA from a variety of bacterial species. The concentration of the genomic DNA was determined using a Thermo Scientific NanoDrop 2000c UV-Vis Spectrophotometer. Standard curves were generated by amplification of serial dilutions of DNA in DNase-free water. PCR reaction efficiencies for the standard curves were between 90% and 105% and all regression coefficients (R^2^) were above 0.98.

### 2.6. MIC for Vancomycin Resistant

Confirmed enterococci (*n* = 1111) were grown on TSA at 37 °C for 24 h and then transferred to 5 mL THB and incubated at 37 °C for 18 h. Susceptibility to vancomycin was tested using an assay in which THB (48 µL) with esculin (1 g/L), FAC (0.7 g/L) and vancomycin (0 mg/L, 4 mg/L or 32 mg/L; Bio Basic, Ontario, Canada) were added to each well in a 96-well plate (Ramquest Technologies, Plano, TX, USA). Isolates (2 µL) and the plates were incubated at 37 °C for 18 h. Isolates that grew at between 4 and 32 mg/L of vancomycin were considered to exhibit intermediate resistance, whereas those that grew at >32 mg/L vancomycin were considered resistant. Reference strains of *E. faecium* ATCC 700221 (MIC ≥ 32 mg/L), *E. faecalis* ATCC 51299 (MIC ≥ 4 mg/L) and *E. faecalis* ATCC 29212 (susceptible) were used as controls. A total of 308 VRE isolates (MIC ≥ 4 mg/L) were identified.

### 2.7. Speciation of Vancomycin-Resistant Enterococci

Speciation of enterococci was completed by sequencing the *gro*ESL gene as described by Zaheer et al. [22] and Sanderson et al. [23]. Briefly, a single bacterial colony from cultures grown overnight at 37 °C on TSA plates was suspended in 100 μlL of ddH_2_O, heat-lysed at 95 °C for 5–10 min and centrifuged at 10,000× *g* for 5 min. From the supernatant, 2 μL was used as a template in a 50 μL PCR reaction containing 200 nM of forward (ENT-ES-211-233-F; 5′-GHACAGAAGTRAAATAYGAAGG-3′) and reverse primers (ENT-EL-74-95R; 5′-GGNCCTAABGTHACTTTNACTG-3′) in a Phusion Flash High-Fidelity PCR Master Mix (Thermo Fisher Scientific). The PCR was performed using an Applied Biosystems SimpliAmp^tm^ Thermocycler with an initial denaturation step at 95 °C for 5 min, followed by 30 cycles of denaturation at 94 °C for 30 s, annealing at 51 °C for 30 s and an extension at 72 °C for 20 s. Aliquots (5 μL) of PCR products were electrophoresed on a 1.5% agarose gel containing a final concentration of 0.2–0.5 µg/mL of ethidium bromide (BioRad). Generated amplicons ranged from 185 to 226 bp [23]. Products were sequenced by Genome Quebec (Montreal, QC, Canada) and compared to known sequences for *Enterococcus* species using NCBI BLASTn.

### 2.8. Antibiotic Susceptibility Testing

Antimicrobial susceptibility of the 308 isolates was determined using disk diffusion according to CLSI [24] and EUCAST ECOFFs [25]. Isolates were grown on Brain Heart Infusion agar (Dalynn Biologicals Inc., Calgary, AB, Canada) at 37 °C for 24 h. This was followed by the preparation of cell suspensions with OD_625_ of 0.160–0.180 in 0.85% saline. The disk diffusion susceptibility test was performed using Mueller-Hinton agar (Dalynn Biologicals Inc.), with the antimicrobials and resistance breakpoints outlined in Table S3. *Staphylococcus aureus* ATCC 25923 and *Enterococcus faecalis* ATCC 29212 were used as reference strains. Zones of inhibition were determined using a BioMic V3 imaging system (Giles Scientific, Santa Barbara, CA, USA).

### 2.9. Data Analysis

Microbiological sampling results were converted to average CFU per 100 mL. The qPCR results were converted to average target gene copy numbers per 100 mL of wastewater. The average enterococcal and VRE loads per inhabitant were calculated using the colony counts of total enterococci or of VRE and the reported population of the entire municipality (117,660 inhabitants). Average values and standard deviation were obtained using values from CFUs and DNA extracts tested in triplicate (at different dilutions). Relative abundance of each gene was calculated by dividing the absolute quantities of each gene with the quantity of 16S rRNA gene and *t*-tests were completed using the R Statistical Platform version 3.4.3 [26].

Removal efficiencies were calculated using the CFU/100mL of enterococci and VRE in the PE relative to the FE in the two WWTPs. The removal efficiency of enterococci and VRE in the two WWTPs was determined using the microbiological sampling data rather than the qPCR results given the high specificity of the qPCR assay for *E. faecalis* and *E. faecium*, which would likely exclude environmental *Enterococcus* spp. Statistical analysis was completed using R Statistical Platform version 3.4.3. Metadata from each sample was analyzed by principal component analysis (PCA), using Past3 (University of Oslo, Oslo, Norway) [27], to determine the factors that correlated with the variance seen among samples. Missing data was supported by column average substitution and values below the detection limit were designated as 0. Figures for the PCA were generated either by Past3 or using the analysis from Past3 and the Chart Builder feature in IBM SPSS Statistics 24 package. The primary and FE metadata were analyzed separately.

A chi-square test was performed using R Statistical Platform version 3.4.3 [26] to determine the differences in the prevalence of species and resistances to particular antimicrobials. A 12-digit code was generated for each isolate to represent their susceptibility to each antimicrobial with “2” indicating resistance, “1” indicating intermediate resistance and “0” indicating susceptibility to each antimicrobial. The codes and isolates were clustered using the unweighted pair group method with arithmetic mean (UPGMA) of group resistances to antimicrobials based on their occurrence in the same isolate. This was used to determine the relationship between resistance phenotypes in terms of possible cross- and co-resistance [28,29]. The Hamming distance [30] between two 12-digit codes was designated as the number of positions at which the corresponding symbols (“2”, “1” and “0”) differ, and was calculated for all isolates. Clustering was assessed using the Hamming distance function of the e1071 package in R version 3.4.3 [31]. Dendrograms of the heatmaps were generated by applying hierarchical clustering with complete linkage. Heatmaps illustrating these analyses were generated for the FE and PE isolates of both WWTPs using the heatmaply package version 0.14.121 [32] in R version 3.4.3.

The average number of prescriptions and units sold for each antimicrobial class (per month) by community pharmacies was calculated for the entire municipality and compared to the prevalence of resistance in wastewater enterococci for each class of antimicrobial. The amount of antimicrobial prescribed and sold within the service areas could not be ascertained because the purchase of antimicrobials within one service area does not guarantee their use within that service area. The average number of prescriptions and units sold per month by community pharmacies was calculated for the sampling period and plotted against the number of isolates resistant to each antimicrobial class. The number of prescriptions and units of antimicrobial sold in hospitals and other clinical settings was unavailable.

## 3. Results

### 3.1. Microbiological Quantification of Enterococci and Vancomycin-Resistant Enterococci

The average enterococci load per inhabitant per day (in CFU) for the BAF system was estimated as 1.88 × 10^8^ for the PE and 1.06 × 10^5^ for the FE. The average enterococci load per inhabitant per day (in CFU) for the CAS system was estimated as 2.18 × 10^8^ for the PE and 2.74 × 10^6^ for the FE. The average VRE load per inhabitant per day (in CFU) for the BAF system was estimated as 5.81 × 10^7^ for the PE and 3.98 × 10^4^ for the FE, whereas for the CAS system, it was estimated as 6.14 × 10^7^ for the PE and 6.81 × 10^5^ for the FE.

There was no difference between the amounts of enterococci (*p* = 0.233) or VRE (*p* = 0.113 and 0.097) detected in either WWTP (Table 1). There were fewer (*p* < 0.001) VRE and enterococci isolated from the FE compared to the PE. The relative quantities of VRE isolated did not differ between treatment plants (Table 1). The removal efficiency for enterococci (*p* < 0.001) and low level VRE (*p* < 0.001) for BAF system was greater than that of CAS system.

The average removal efficiencies for enterococci were 99.8% for the BAF system and 98.2% for the CAS System (Table 1). The removal efficiencies for enterococci ranged from 99.2% to 99.9% for the BAF system and from 93.0% to 99.9% for the CAS system. The average removal efficiencies for VRE (MIC = 4 mg/L) were 99.9% for the BAF system and 97.8% in the CAS system. The average removal efficiencies for high MIC (32 mg/L) were 99.6% for the BAF system and 96.8% for the CAS system. The removal for VRE was the same as for enterococci in the BAF system and greater than (*p* < 0.001) the CAS system, which ranged for 84.6–99.9%.

### 3.2. Molecular Quantification of Enterococci, Vancomycin Resistance (vanA) and Class I Integrons

The absolute quantities of bacteria, enterococci and class 1 integrons were similar regardless of source. The absolute quantity of copies of the vancomycin resistance gene (vanA) was greater (*p* = 0.003) in the PEs of both WWTPs compared to the FEs (Table 2). The relative quantities of enterococci were greater (*p* = 0.038) in the CAS than the BAF system and greater (*p* = 0.043) in the PE compared to the FE. The relative quantities of vancomycin resistance genes and class 1 integrons were similar regardless of source.

### 3.3. Antimicrobial Susceptibility Testing

Enterococcal isolates (*n* = 1111) collected before the filters were transferred into selective media containing 4 mg/L vancomycin, resulting in a total of 308 isolates being recovered. Results were reported as VRE resistant to each antimicrobial based on disc diffusion as a percentage of the total number of enterococci that were isolated. The detection of resistance to vancomycin was higher in the broth-based assay as compared to the disc susceptibility test, suggesting that the broth-based test detected isolates with intermediate resistance that were not detected in the disc susceptibility assay. Isolates with a MIC equal to or greater than 4 mg/mL were considered to be VRE.

Disk diffusion susceptibility testing identified intermediate or resistance to at least one antimicrobial in 93.3% of the VRE isolates. Many of the susceptible isolates (*n* = 21) were *E. casseliflavus*/*E. gallinarum* (*n* = 17), which are intrinsically resistant to vancomycin. The majority of VRE were *E. faecalis* (*n* = 151) and *E. faecium* (*n* = 94), followed by *E. casseliflavus*/*E. gallinarum* (*n* = 58), *E. hirae* (*n* = 3), *E. mundtii* (n = 1) and *E. saccharolyticus* (*n* = 1; Table 3). The isolates collected from the biomass of the two WWTPs were predominately *E. faecalis* (*n* = 8) followed by *E. casseliflavus*/*E. gallinarum* (*n* = 2) and *E. hirae* (*n* = 1) and shared AMR profiles similar to that of PE and FE isolates. Resistance to 3 or more antimicrobials was detected in 23.4% (*n* = 72) of enterococci. The majority of the multidrug-resistant enterococci were *E. faecalis* (*n* = 46) followed by *E. faecium* (*n* = 25), with the prevalence of multidrug resistance being similar between these species (Table S4). There were more (*p* = 0.001) multidrug resistant enterococci isolated from BAF (*n* = 14) than CAS FE (*n* = 0), with similar levels of multidrug resistant enterococci in the PE of both systems. The relative abundance of resistance to each antimicrobial differed depending on species (Table 4). Resistance to all antimicrobials, except gentamicin (*p* = 0.406) and streptomycin (*p* = 0.123), differed based on the species of enterococci. *E. faecium* was more often (*p* = 0.013) resistant to vancomycin, ampicillin, erythromycin, nitrofurantoin, levofloxacin (*p* < 0.001) and streptomycin as compared to other species. In contrast, *E. faecalis* were more frequently (*p* < 0.001) resistant to doxycycline (*p* < 0.001), linezolid (*p* = 0.005) and quinupristin/dalfopristin than other species. Resistance to teicoplanin, ampicillin, erythromycin, levofloxacin, quinupristin/dalfopristin and streptomycin was also detected in *E. casseliflavus* and *E. gallinarum*. No resistance was detected in *E. mundtii*, *E. hirae* or *E. saccharolyticus*, and none of the isolates were resistant to tigecycline. While the species distribution in the PEs was similar, the FE from BAF had a higher (*p* = 0.047) relative abundance of *E. faecalis* and *E. faecium*, than the CAS FE.

**Figure 2:**
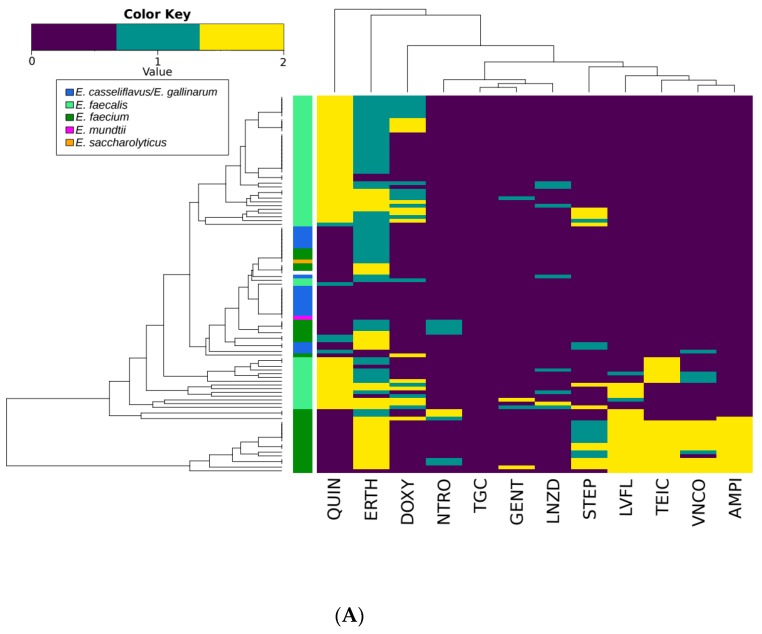

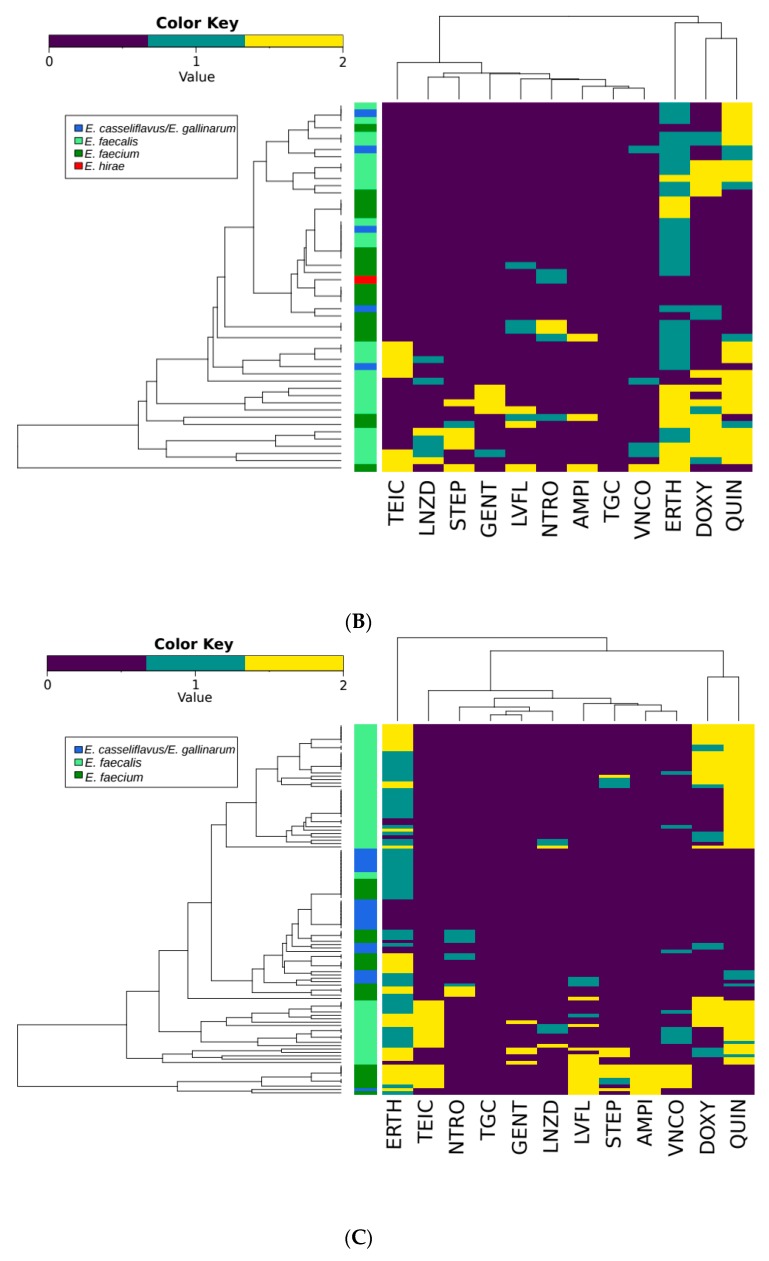

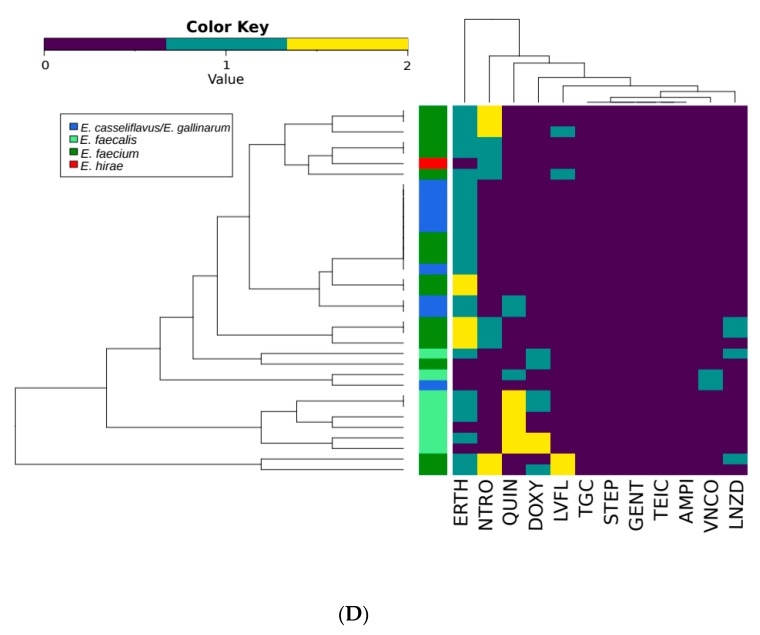
The resistance profiles of the *Enterococcus* spp. indicating the isolates, which are resistant (yellow), intermediate resistant (aqua) and susceptible (dark blue) to antimicrobials. The tree on top of the heatmap depicts the clustering of antimicrobials to which resistance occurs in the same isolates from primary (**A**) and final effluent (**B**) of the biological aerated filter (BAF) and the primary (**C**) and final effluent (**D**) of the conventional activated sludge (CAS) system. The dendrogram on the side of the heatmap shows the phylogenetic relationship among isolates. VNCO, vancomycin; TEIC, teicoplanin; AMPI, ampicillin; DOXY, doxycycline; ERTH, erythromycin; NTRO, nitrofurantoin; GENT, gentamicin; LNZD, linezolid; LVFL, levofloxacin; QUIN, quinupristin/dalfopristin; STEP, streptomycin.

There were 110 different AMR profiles identified in the 308 enterococcal isolates (Table S5). The majority of AMR profiles were unique to the PE or FEs of one of the WWTPs (Figure 1), with 76 profiles only occurring in isolates from a single source. Only seven profiles were shared between the primary and FE of both WWTPs suggesting changing profiles as isolates pass through wastewater treatment process. The three most common profiles were intermediate resistance to erythromycin (*n* = 40), intermediate resistance to erythromycin, resistance to quinupristin/dalfopristin (*n* = 24), intermediate resistance to erythromycin and resistance to doxycycline and quinupristin/dalfopristin (*n* = 15; Table S5). The individual resistance phenotypes of isolates clustered similarly in the PE from both WWTPs, but differed in the FEs with no isolates exhibiting resistance to ampicillin, gentamicin, streptomycin or tigecycline (Figure 2). The most common phenotypes across all systems included intermediate resistance or resistance to erythromycin, doxycycline and quinupristin/dalfopristin. Vancomycin and ampicillin resistance was also common in these isolates. Co-occurrence of nitrofurantoin and linezolid resistance was also frequently observed in these isolates. Resistance to gentamicin and streptomycin was rarely detected in the same isolate.

The prevalence of resistance to teicoplanin (*p* = 0.034), ampicillin (*p* = 0.040), nitrofurantoin (*p* = 0.004), quinupristin/dalfopristin (*p* = 0.038) and streptomycin (*p* = 0.034) differed between influent and effluent, but not between BAF and CAS systems (Table 5). The prevalence of teicoplanin (*p* = 0.041), nitrofurantoin (*p* = 0.012), levofloxacin (*p* = 0.014) and streptomycin (*p* = 0.047) differed in effluent from the BAF vs. CAS system. In the BAF system, there were fewer isolates resistant to levofloxacin, but more (*p* = 0.018) intermediate resistance to this antimicrobial. In the CAS system, there were fewer isolates resistant to teicoplanin (*p* = 0.012) and quinupristin/dalfopristin (*p* = 0.004), but more isolates resistant to nitrofurantoin (*p* = 0.001). Comparing the isolates from the FEs of both systems, there were more isolates resistant to teicoplanin (*p* = 0.037), doxycycline (*p* = 0.039), nitrofurantoin (*p* = 0.039) and quinupristin/dalfopristin (*p* = 0.019) from the CAS system, whereas the prevalence of isolates with resistance to these antimicrobials did not differ in the PEs.

### 3.4. Principal Component Analysis of Chemical and Environmental Factors

Table 6 summarizes the average values for all of the chemical and environmental factors associated with the wastewater in both WWTPs. The principle component analysis shows how different the samples from each wastewater treatment process and each sample site within the process are compared to one another. It also provides some indication of the factors allowing the BAF system to release fewer enterococci back into the environment. In both the PE and FE, the most heavily weighted factors are alkalinity and total suspended solids (TSS) content of the wastewater.

The abundance of enterococci and VRE were similar in the PEs of the two WWTPs, while the land use within the service areas of the two WWTPs differed (Figure S1). This would suggest that the origin of the wastewater may not affect the abundance of enterococci in the PE of the WWTPs or that there was no difference between the service areas. Increased land cover for commercial, industrial, residential and other development related uses were positively correlated with most of the variance (PC1, 79%), while environmental protection areas were negatively correlated with variance in the PE samples. Land use in the service areas may have influenced the chemistry of the PE (Figure S2).

In the PE, the samples from each WWTP clustered together based on a combination of the first two principal components (PCs; Figure S2) with PC1 accounting for most of the variance among samples. PC1 (62.6%) had positive correlations (*p* > 0.05) with cobalt, zinc, total suspended solids (TSS), total phosphorus, total Kjeldahl nitrogen (TKN), total ammonia and unionized ammonia content of the wastewater. For the FE, the samples from each WWTP clustered based on the first two PCs as well (Figure S3). PC1 (65.1%) correlated with copper (*p* > 0.05), alkalinity, pH, TSS, total ammonia, unionized ammonia and nitrate content of the wastewater. Correlations with copper and nitrate were negative, while the other parameters had positive correlations with PC1. This suggests that these factors have an impact on WWTP performance and the survival of enterococci.

## 4. Discussion

While enterococci primarily reside in the intestinal tract of animals, they can be abundant in wastewater and tend to be more prominent than other streptococci [33]. The persistence of enterococci in water and other environments make *Enterococcus* spp. a suitable indicator of fecal contamination [34]. The average loads of enterococci per inhabitant per day were ten-fold lower in the PE and up to 10,000-fold lower in PE compared to a study of 14 municipal WWTPs in Portugal [28]. The discrepancy in these values for the FE could be due to differences in effluent disinfection protocols between the WWTPs in this study, which both used chlorination, whereas the WWTPs examined by Martins da Costa et al. [28] and Ferriera et al. [7] lacked tertiary treatment.

A factor that can differ between studies is the methodologies used to quantify, isolate and identify enterococci. The use of bile azide agar [35], Slanetz and Bartley agar ([28,29] and this study) or m-Enterococcus agar [36] may influence isolate recovery as *Enterococcus* spp. vary in β-glucosidase activity, which influences the phenotypic characteristics (degree of red pigment formation in the colony) used to identify enterococci colonies [37,38]. Additionally, phenotypic methods [39,40] and amplicon sequencing using additional targets can be unreliable and result in the exclusion of some environmental *Enterococcus* spp., particularly *E. casseliflavus* and *E. gallinarum* as compared to groESL amplicon sequencing [41,42,43]. Consequently, method selection could result in the over representation of some enterococci species and the under representation of others.

Discrepancies in speciation could also be due to the use of the groESL and 23S loci for the identification of enterococci as opposed to phenotypic methods, like the API 20 Strep test (BioMerieux, Marcy-l’Étoile, France), used by Martins da Costa et al. [28] or the use of partial 16S rRNA sequences [43]. In a comparison of biochemical and genotypic methods for the identification of wastewater enterococci, the *gro*ESL loci was found to be ideal [23], with the 23S rRNA loci considered the gold standard for quantifying enterococci using qPCR [19]. Additionally, studies typically do not correct for false positive and negatives produced through selective media [7]. Finally, the frequency of vancomycin resistance and the species composition of the *Enterococcus* spp. isolated from wastewater can differ geographically, complicating comparisons among studies [33]. The removal efficiencies documented in this study are comparable to other studies [35]. Neither WWTP was able to eliminate all AMR enterococci, but did lower them as these systems are designed to retain biosolids and have a disinfection step to reduce enterococci and other microorganisms in the final effluent. The removal efficiency of the CAS system was more variable (93.0–99.9%) than the BAF system (99.2–99.9%), possibly related to the age and decline in efficiency of the WWTPs as well as the treatment process. The CAS system is now in the process of being upgraded to a BAF system [36].

The molecular quantification of enterococci and subsequent species identification in this study suggests a shift in the relative proportion of the *Enterococcus* species in the FEs. The primers used for qPCR were more specific to *E. faecium* and *E. faecalis* and thus were not suitable for defining the fate of all enterococci species during wastewater treatment [19]. The relative abundances of the vancomycin resistance gene (vanA; *p* = 0.070) and class I integrons (*p* = 0.917) did not differ between WWTPs or effluents. Additionally, fewer samples were collected for qPCR analysis than for microbiological analysis, a factor that could also account for some discrepancies.

In agreement with our study, others have found *E. faecium* and *E. faecalis* to be the predominant enterococci in urban wastewater [44]. Other species also detected in hospital and urban wastewater include *E. hirae*, *E. durans*, *E. casseliflavus*, *E. gallinarum* and *E. flavescens* [33]. However, studies of the distribution and characterization of enterococci in wastewater are scarce compared to studies using other indicators of fecal contamination [45], limiting conclusions about their utility and role in the dissemination of AMR [7]. In multiple studies, *E. faecium* has been the dominant *Enterococcus* spp. isolated from WWTPs [33,35,46,47], while others align with our study where *E. faecalis* was identified as the dominant species [28,36,48]. Additionally, we recovered *E. faecalis* from the activated sludge of the CAS system, contrary to Schwartz et al. [49] who were unable to isolate this bacterium from biomass originating from a hospital, urban wastewater or surface water. Factors that can influence the distribution of *Enterococcus* spp. in wastewater include geographical differences [50,51], variation in land use, diet (Blanch et al., 2003) [33], seasonal influences and differences in study methodologies. For instance, in many Canadian surveys, *E. faecalis* is more dominant in waters flowing from areas used for livestock production, whereas *E. faecium* may be more common in urban wastewater [50,51].

In this study, analysis of land use within the service areas of the WWTPs was undertaken in an attempt to generate insight into the impact of the origin of the wastewater on the ecology of enterococci. The quantity of enterococci and VRE, prevalence of species and resistance to individual antimicrobials in the PE of the WWTPs were similar despite differences in the service area of the WWTPs. This would suggest that the origin of the wastewater may not have an impact on these factors and may not be a confounding factor in this analysis. The AMR profiles of the enterococci differed between the PE of the two WWTPs, with the majority of profiles unique to each WWTP. In PE, the enterococci could be considered a mosaic of strains, which represent enterococci from a variety of sources, in which AMR profiles of an individual isolate can differ. However, the overall prevalence of resistance and the species present did not differ in PE between the two WWTPs.

The prevalence of enterococci and VRE was associated with numerous chemical components of the wastewater while environmental factors were only weakly associated. In the PE and FE, alkalinity and total suspended solids were heavily weighted factors in the principal component analysis. The average values for these parameters were almost double that in the CAS as compared to the BAF system, suggesting that samples with higher alkalinity and TSS content contain more enterococci. Alkalinity is a measure of the ability of the water to neutralize acid or to absorb hydrogen ions. Wastewater microorganisms grow best at a neutral to slightly alkaline pH of 7–8 [52]. TSS is a measure of particulate dry weight obtained by separating particles from a water sample using a filter, similar to the measurement of turbidity. TSS not only impacts the alkalinity of the water, but also provides substrates for microorganisms to attach to, contributing to their persistence in the waste stream [53]. This results in more enterococci being released into receiving water as indicated by the difference in removal rate of enterococci and VRE between the WWTPs. Higher TSS in the FE is a sign of poor efficiency in the system and the older CAS system had higher TSS resulting in high enterococci numbers in the FEs. Increased TSS could also result in closer interactions among bacteria, possibly facilitating the spread of AMR. For example, tetracycline resistance rates were amplified by increases in TSS in wastewater [54].

Excessive phosphorus and nitrogen can cause eutrophication, and an excess of nutrients can cause ecological changes, like algal blooms [55]. So, WWTPs are tasked with reducing the amount of phosphorus and nitrogen released in effluent. The amount of soluble phosphorus was greater in the BAF PE and the amount of total phosphorus was greater in the CAS PE (Table 6). Nitrogen is measured in a variety of forms in the FE including total Kjeldahl nitrogen (TKN) and total ammonia and nitrate (Table 6), which differed between the two WWTPs. The measures for nitrogen, particularly total ammonia can be considered measures of the effectiveness and management of the chlorination of treated wastewater. For instance, high total ammonia content affects the efficacy of chlorination as a disinfection method and corresponds with a decline in the removal of AMR bacteria from the FE during chlorination [56]. These same measures can be used to determine the amount of nitrification that has occurred during the treatment process, which also impacts the alkalinity of the wastewater [57]. The BAF system had lower ammonia levels and fewer enterococci in its FE, suggesting that disinfection was more efficient in this system compared to the CAS system.

Resistance to copper stress has been associated with co-selection of genetic determinants for copper resistance and antimicrobial resistance [58]. Copper can be found in wastewater in high concentrations and the removal of copper from wastewater is incentivized both for environmental and for financial reasons given its value in industrial applications [59]. Copper can also encourage the acquisition and development of cross-resistance between it and some antimicrobials including vancomycin resistance in enterococci [60].

A shift from *E. hirae* to *E. faecium* during wastewater treatment was observed by Ferriera da Silva et al. [7]. They suggested that this could be due to the higher intrinsic tolerance of *E. faecium* to environmental stress as compared to *E. hirae* [7,61]. The BAF system includes polystyrene media carriers in addition to suspended solids, which increases the surface area available for biofilm formation. In contrast, the CAS system does not incorporate media carriers. Biofilm production in *Enterococcus* spp. has been associated with pathogenicity, particularly with endocarditis. *E. faecalis* isolates from clinical and fecal sources more readily form biofilms than *E. faecium* [62]. The increased relative abundance of *E. faecalis* in the FE of the BAF system in our study could reflect the superior ability of *E. faecalis* to form biofilms.

The detection of multiple resistances in VRE was consistent with previous studies [35,36], although the prevalence varied. The amount of multidrug resistance observed differed among species with resistance to three or more antimicrobials detected in 30.5% of *E. faecalis* (46/151), 25.0% of *E. faecium* (25/94) and only 1.7% of *E. casseliflavus*/*E. gallinarum* (1/59) isolates. Multidrug resistance in *E. faecium* and *E. faecalis* is more concerning than in other species given their role as etiological agents of disease and their exposure to high concentrations of antimicrobials in the digestive systems of humans and livestock [20,47]. In this study, multidrug resistance was most prevalent among *E. faecalis* and its prevalence did not decline (23.8–27.5%) in the BAF system, but did (28.2–0%) in the CAS system. This difference could be indicative of the parameters of the treatment process. For example, Martins da Costa et al. [29] found no difference in the resistance rates between enterococci isolated from poultry slaughterhouse waste influent or treated effluents. Luczkiewicz et al. [35] found that fecal indicators exhibiting multidrug resistance were positively selected for during treatment in an activated sludge WWTP. The distribution of unique AMR phenotypic profiles could depend not only on the characteristics of enterococci arriving in the influent, but also reflect the loss or acquisition of genes in WWTPs [63]. Additionally, the AMR phenotypic profiles of the PE and FE of the two WWTPs differed based on Hamming distances [30], which also suggests the acquisition and loss of resistance phenotypes during treatment.

The clustering of ampicillin and vancomycin in the BAF FE and PEs was consistent with previous studies, [28], suggesting that ampicillin resistance could be linked to vancomycin resistance. This phenotype may arise as a result of mutations in penicillin binding proteins or adaptations to maintain cell wall function in the presence of glycopeptides [15]. This clustering was not observed in isolates from the CAS FE as none were resistant to ampicillin. Other antimicrobials that cluster due to possible cross-resistance or co-selection include levofloxacin with streptomycin; erythromycin with doxycycline and quinupristin/dalfopristin. These phenotypes often arise due to acquisition of a single mechanism that confers resistance to multiple antimicrobials. For example, levofloxacin and streptomycin resistance can be plasmid-mediated through determinants such as AAC(6′)-Ib-cr, which confer resistance to aminoglycosides and fluoroquinolones or due to efflux pumps that confer resistance to other unrelated antimicrobials [15,64].

Erythromycin and doxycycline resistance can also be co-selected [28,29] and determinants, such as ermB can confer cross-resistance between streptogramins (quinupristin/dalfopristin) and macrolides [15]. Although a linkage between erythromycin and tetracycline has been identified in previous wastewater studies as well as in agricultural and clinical settings [28,29,65], such an association was not apparent in our study. The use of doxycycline as a representative of tetracyclines may account for this as pharmacokinetics differ among tetracyclines, and not all members of the tetracycline class are equally affected by efflux or ribosomal protection mechanisms [66].

Similar to this study, Blanch et al. [33] observed that most of the wastewater isolates with high-level vancomycin resistance (MIC ≥ 32 mg/L) were also resistant to erythromycin. Macrolide resistance could favor the persistence of VRE in the environment due to co-selection for resistance to more widely used antimicrobials, like erythromycin [67]. The high level of resistance to erythromycin and doxycycline in wastewater enterococci coincided with the high number of macrolide and tetracycline prescriptions in the municipality. Ferreira da Silva et al. [7] also observed that macrolide and quinolone resistance in enterococci coincided with the clinical use of these antimicrobials. The prevalence of resistance to the other classes of antimicrobials did not correspond to the number of prescriptions and units of antimicrobials sold. Resistance to penicillins and β-lactams was low even though the amount of units sold and prescriptions associated with these antimicrobial classes was among the highest. Penicillin can be degraded in aquatic environments, reducing its ability to promote AMR in wastewater [68]. The consumption data for quinupristin/dalfopristin was not available, but 45.1% of the isolates were resistant, as *E. faecalis* is intrinsically resistant to these antimicrobials.

In this study, community sales of antimicrobials were not a good indicator of enterococci resistance in wastewater. During the sampling period, the average number of prescriptions for vancomycin per month was 27.2 and the average number of units sold per month in community pharmacies within the municipality was 436.8. Over the course of the sampling period, spikes in vancomycin use did not coincide with spikes in the abundance of VRE in the PE or FEs of WWTPs. The rate at which an antimicrobial is metabolized by humans and animals (or in wastewater) can differ depending on a variety of factors including the class of antimicrobial and the wastewater treatment process [69]. While concentrations of antimicrobial residues in hospital or retirement home effluent can be high, the downstream effluent concentrations are often low, making it likely that their contribution to selective pressure for multi-drug resistant enterococci is low [49]. However, this does not eliminate the possibility that higher concentrations of antimicrobials may accumulate in sediments and biomass in wastewater, exerting selective pressure for multi-drug resistance, especially quinolones [45]. Doxycycline and erythromycin concentrations in biosolids can be in the parts per million (ppm) and parts per billion (ppb) range, respectively [70].

Enterococci are intrinsically resistant to low concentrations of aminoglycosides, such as gentamicin and streptomycin. In this study, streptomycin resistance was more common in *E. faecium* and gentamicin resistance was more common in *E. faecalis.* Compared to gentamycin, streptomycin is rarely used and there is no cross-resistance with other aminoglycosides, except for when resistance is caused by impermeability (15). The mechanism for gentamicin resistance cannot be determined solely by phenotypic testing [66]. The lack of cross-resistance between the two aminoglycosides would suggest differing resistant mechanisms in the wastewater isolates.

Nitrofurantoin is a drug of last resort for the treatment of VRE urinary infections [71]. Nitrofurantoin resistance was more common in the FE of the CAS system and was most often associated with *E. faecium*. Resistance to nitrofurantoin requires both the acquisition of genes for plasmid-mediated efflux pumps and mutations in genes for oxygen-insensitive nitroreductases and riboflavin/flavin mononucleotides [72,73]. Resistance to nitrofurantoin in *E. coli* isolated from water and sediments has started to gain attention and some suggestions for environmental and clinical surveillance of nitrofurantoin resistance have been proposed [73].

While it could be argued that these observations are the result of sample size, other studies have suggested that culture collections generated from WWTPs are representative of the overall population of enterococci in wastewater [33]. Given that the prevalence of AMR correlates with the prevalence of a particular species, the ecology of enterococci in different wastewater processes should be investigated further if the impact of treatment process on the prevalence of clinically important *Enterococcus* spp. and AMR in those organisms is to be elucidated. The similarities between the PEs and dissimilarities in the FEs would suggest that these changes are a result of treatment and not just a function of the influent composition. The more subtle differences in AMR phenotype in the wastewater suggest shifts in the enterococcal populations during the treatment process, especially given that the relative abundances of vanA and Class I integrons did not differ between WWTPs or effluents.

## 5. Conclusions

WWTPs can be a point source and a point of control for environmental AMR including multidrug-resistant enterococci. In summary, the BAF system outperformed the CAS system for the removal of enterococci. The removal of VRE is proportional to the removal of the total population of enterococci. Neither treatment process could efficiently eliminate AMR enterococci. The abundance of enterococci may not be impacted by the origin of the influent given that the abundance of enterococci in the PE of the two WWTPs was similar. However, land use may impact the overall AMR profiles expressed by wastewater enterococci and could also impact the survival of enterococci through the wastewater treatment process. The sale of a class of antimicrobial within the community was not necessarily reflected in the prevalence of resistance in wastewater enterococci isolates, with the exception of macrolides and tetracyclines. The use of vancomycin in the community did not correlate with spikes of VRE in wastewater. Comparing the species and prevalence of resistance in enterococci in the primary and FEs, the BAF system decreased the prevalence of levofloxacin resistance and the CAS system increased nitrofurantoin and decreased quinupristin/dalfopristin and teicoplanin resistance in enterococci. Enterococci populations in the wastewater should be explored further to determine the impact of the origin of the influent on relative species composition and AMR profiles of wastewater enterococci. Factors that differentiate the samples from each WWTP are the alkalinity, pH, TSS content, phosphorus and nitrogen content. Secondary factors include the copper content of the wastewater with temperature and cumulative precipitation being tertiary factors. The individual impact of these factors on the survival and abundance of VRE in wastewater merits further investigation.

## Figures and Tables

**Table 1:** Microbiological counts of vancomycin resistant enterococci and enterococci in the primary and final effluents from biologically aerated filter (BAF) and conventional activated sludge (CAS) wastewater treatment plants.

	Biological Aerated Filter		Conventional Activated Sludge		SEM	*p* Value *	
	Primary Effluent	Final Effluent	Primary Effluent	Final Effluent		WWTP	Effluent
Absolute Quantities (CFU/100mL)							
Total *Enterococcus* ×10^3^	72.054	109.764	0.0718	1.381	8.638	0.233	<0.001
Low Level VRE ×10^3^	17.830	28.428	0.0169	0.356	1.820	0.113	<0.001
High Level VRE ×10^2^	26.482	57.685	0.0457	0.226	5.264	0.097	<0.001
Relative Quantities (%)							
Low Level VRE	35.779	31.856	31.697	28.117	1.622	0.255	0.235
High Level VRE	9.869	11.637	8.535	4.113	1.266	0.673	0.124
Removal Efficiency (%)							
Total *Enterococcus*	99.837		98.210		0.218	<0.001	
Low Level VRE	99.850		97.777		0.387	<0.001	
High Level VRE	99.588		96.817		0.736	0.059	

Low level VRE are enterococci with an MIC in liquid broth (THB) supplemented with vancomycin at 4 to 32 mg/L. High Level VRE are enterococci with an MIC in liquid broth (THB) supplemented with vancomycin of >32 mg/L. * *p* value by standard t-test.

**Table 2:** Quantification of enterococci, total bacteria, class I integrons and vanA genes in the primary and final effluents from biologically aerated filter (BAF) and conventional activated sludge (CAS) wastewater treatment plants using quantitative PCR.

	Primary Effluent		Final Effluent		SEM	*p* Value *			
	BAF	CAS	BAF	CAS		WWTP	Effluent	Primary Effluent	Final Effluent
**Absolute Quantities (copies/100mL)**									
Total Bacteria (16S rRNA) ×10^4^	3.628	24.727	1.189	2.250	5.451	0.315	0.258	0.524	1.000
Total *Enterococcus* (23S rRNA) ×10^3^	0.0163	13.836	0.032	0.066	3.348	0.312	0.305	0.478	1.000
Vancomycin resistance gene (vanA)	32.673	16.195	8.243	3.002	3.280	0.099	0.003	0.203	0.919
Class I integrons (IntI-1) ×10^4^	6.019	132.635	2.194	3.559	22.174	0.153	0.134	0.168	1.000
Relative Quantities (%)									
Total *Enterococcus* (23S rRNA) ×10^−2^	0.430	2.297	0.966	1.297	0.253	0.038	0.570	0.043	0.988
Vancomycin Resistance Gene (vanA) ×10^−4^	0.109	0.041	0.189	0.036	0.030	0.070	0.545	0.853	0.290
Class I integrons (IntI-1) ×10^2^	2.002	20.547	21.553	5.432	5.716	0.917	0.849	0.667	0.756

BAF, biological aerated filter; CAS, conventional activated sludge; SEM, standard error of the mean, WWTP, wastewater treatment plant; * *p* value by standard t-test.

**Table 3:** Species and numbers of enterococci isolates from primary effluent, final effluent and biomass from the biological aerated filter (BAF) and conventional activated sludge (CAS) wastewater treatment plants.

Sample Types	Species	% Isolates	Samples Types	Species	% Isolates
BAF Primary Effluent (*n* = 101)	*E. faecium* (*n* = 30)	29.7	CAS Primary Effluent (*n* = 110)	*E. faecium* (*n* = 28)	25.5
	*E. faecalis* (*n* = 51)	50.4		*E. faecalis* (*n* = 58)	52.7
	*E. casseliflavus/E. gallinarum* (*n =* 18)	17.8		*E. casseliflavus/E. gallinarum* (*n* = 24)	21.8
	Other (*n* = 2)	2		Other (*n* = 0)	0
BAF Final Effluent (*n* = 51)	*E. faecium* (*n* = 19)	37.3	CAS Final Effluent (*n* = 35)	*E. faecium* (*n* = 17)	48.6
	*E. faecalis* (*n* = 26)	51		*E. faecalis* (*n* = 8)	22.9
	*E. casseliflavus/E. gallinarum* (*n* = 5)	9.8		*E. casseliflavus/E. gallinarum* (*n =* 9)	25.7
	Other (*n* = 1)	2		Other (*n* = 1)	2.9
BAF Biomass (*n* = 1)	*E. faecium* (*n* = 0)	0	CAS Biomass (*n* = 10)	*E. faecium* (*n* = 0)	0
	*E. faecalis* (*n* = 0)	0		*E. faecalis* (*n* = 8)	80
	*E. casseliflavus*/*E. gallinarum* (*n* = 0)	0		*E. casseliflavus*/*E. gallinarum* (*n* = 2)	20
	Other (*n* = 1)	100		Other (*n* = 0)	0

BAF, biological aerated filter; CAS, conventional activated sludge.

**Table 4:** Prevalence of resistance phenotypes in each species with the *p* values in prevalence for differences between species ^a^.

		VNCO	TEIC	AMPI	DOXY	ERTH	NTRO	GENT	LNZD	LVFL	QUIN	STEP
*E. faecalis*	151	0 15	30	0	56 34	42 95	0 0	9 3	5 20	10 3	137 7	137 7
*E. faecium*	94	20 1	22	26	6 3	44 41	12 23	1 0	0 3	30 7	1 5	1 5
*E. casseliflavus/E. gallinarum*	58	0 4	1	1	0 1	4 32	0 0	0 0	0 1	1 2	1 9	1 9
Other *Enterococcus spp.*	5	0 0	0	0	0 1	0 2	0 2	0 0	0 0	0 0	0 0	0 0
Total	308	20 20	53	27	62 58	90 168	12 25	10 3	5 24	41 12	139 21	25 17
^b^*P* values												
*E. faecalis* vs *E. faecium*		<0.0001	0.6187	<0.0001	<0.0001	0.0073	<0.0001	0.062	0.0052	<0.0001	<0.0001	0.0134
All Species		<0.0001	0.016	<0.0001	<0.0001	<0.0001	<0.0001	0.4059	0.0497	<0.0001	<0.0001	0.1227

^a^ Where the number of isolates are displayed in two rows the top number indicates the number of resistant isolates and the bottom number indicates the number of intermediate resistant isolates. ^b^ Chi-sqaure values and *p*-values to determine significance of difference between relative abundances of isolates resistant to each antimicrobial agent. VNCO, vancomycin; TEIC, teicoplanin; AMPI, ampicillin; DOXY, doxycycline; ERTH, erythromycin; NTRO, nitrofurantoin; GENT, gentamicin; LNZD, linezolid; LVFL, levofloxacin; QUIN, quinupristin/dalfopristin; STEP, streptomycin.

**Table 5:** Prevalence of AMR in VRE isolated from primary influent and final effluents of the two WWTPs.

Location		Total	VNCO broth	ABX	VNCO disk	TEIC	AMPI	DOXY	ERTH	NTRO	GENT	LNZD	LVFL	QUIN	STEP
		#	#	R or I	#	#	#	#	#	#	#	#	#	#	#
Biological Aerated Filter (n = 484)	PE	270	101	R	12	21	15	14	33	2	2	1	21	48	11
				I	5			16	51	7	2	7	2	5	11
	FE	214	51	R	1	8	3	14	14	2	4	2	3	23	5
				I	4			6	29	4	1	5	4	5	1
Conventional Activated Sludge (n = 611)	PE	375	110	R	7	21	9	26	35	3	4	2	15	54	9
				I	9			11	59	7	0	5	4	6	5
	FE	236	35	R	0	0	0	2	5	5	0	0	2	6	0
				I	2			5	24	7	0	4	2	3	0
	Total	1095	297	Total R	20	50	27	56	87	12	10	5	41	131	25
				Total I	20			38	163	25	3	21	12	19	17
				*p* values											
				ABX	VNCO	TEIC	AMPI	DOXY	ERTH	NTRO	GENT	LNZD	LVFL	QUIN	STEP
					(Disk)										
				All Four Locations	0.1467	0.034	0.04	0.1008	0.5162	0.004	0.2447	0.5282	0.0793	0.0382	0.0343
				WWTP	0.3924	0.3665	0.1371	0.6744	0.7242	0.3193	0.1968	0.7799	0.5973	0.612	0.0875
				Effluent	0.0501	0.0409	0.0547	0.9974	0.2058	0.0124	0.7236	0.2886	0.0139	0.0516	0.0474
				BAF PE vs FE	0.1016	0.5907	0.177	0.1198	0.7456	0.7577	0.215	0.376	0.0177	0.5224	0.1438
				CAS PE vs FE	0.2623	0.0118	0.1786	0.0618	0.128	0.0012	0.5812	0.2545	0.4053	0.0037	0.085
				BAF FE vs CAS FE	0.6509	0.0373	0.3885	0.0389	0.347	0.0394	0.1618	0.4874	0.9285	0.0188	0.1093

PE, primary effluent; FE, final effluent; R, resistant; I, intermediate resistant; VNCO, vancomycin; TEIC, teicoplanin; AMPI, ampicillin; DOXY, doxycycline; ERTH, erythromycin; NTRO, nitrofurantoin; GENT, gentamicin; LNZD, linezolid; LVFL, levofloxacin; QUIN, quinupristin/dalfopristin; STEP, streptomycin.

**Table 6:** Summary of chemical and environmental metadata for influent and effluent samples collected from biologically aerated filter (BAF) and conventional activated sludge (CAS) wastewater treatment plants. Values are an average for each parameter.

Chemical and Environmental Parameters			Primary Effluent		Final Effluent	
Parameter	Unit	Detection Limit	BAF	CAS	BAF	CAS
Max Temperature	°C	N/A	22.2 ± 5.69			
Min Temperature	°C	N/A	9.30 ± 6.70			
Accumulative Precipitation	mm	N/A	3.77 ± 7.70			
Alkalinity (CaCO_3_, pH 4.5)	mg/L	3	172 ± 16.1	253 ± 17.5	73.6 ± 16.9	143 ± 33.5
pH (25 °C)	pH units	N/A	7.62 ± 0.162	7.45 ± 9.24 × 10^−2^	7.40 ± 0.111	7.61 ± 0.138
CBOD_5_	mg/L	2	57.8 ± 35.7	138 ± 39.2	<2	3.26 ± 2.28
TSS **	mg/L	3	81.9 ± 53.9	165 ± 56.5	33.2 ± 21.7	67.3 ± 68.4
Total P	mg/L	0.01	2.37 ± 0.99	4.09 ± 0.99	0.460 ± 0.15	0.677 ± 0.31
Total Ammonia	mg/L	0.01	13.0 ± 3.58	25.9 ± 6.47	0.820 ± 0.636	14.3 ± 5.87
Ammonia (unionized)	mg/L	0.01	0.150 ± 7.45 × 10^−2^	0.328 ± 0.129	<0.01	0.320 ± 0.264
TKN	mg/L	0.1	17.9 ± 5.88	39.3 ± 8.61	2.27 ± 0.862	16.8 ± 7.89
Nitrite	mg/L	0.1	<0.1	<0.1	0.220 ± 0.237	1.88 ± 0.801
Nitrate	mg/L	0.1	<0.1	<0.1	15.9 ± 3.38	14.0 ± 4.85
Aluminum ***	mg/L	0.01	0.388 ± 0.292	NM	0.982 ± 7.03 × 10^−2^	NM
Arsenic ***	mg/L	5.00 × 10^−4^	<5.00 × 10^−4^	<5.00 × 10^−4^	<5.00 × 10^−4^	<5.00 × 10^−4^
Cadmium ***	mg/L	0.005	<0.005	<0.005	<0.005	<0.005
Chromium ***	mg/L	0.002	1.00 × 10^−2^ ± 9.00 × 10^−3^	<0.002	<0.002	<0.002
Cobalt ***	mg/L	0.005	<0.005	2.00 × 10^−2^ ± 3.40 × 10^−2^	<0.005	<0.005
Copper ***	mg/L	0.002	3.98 × 10^−2^ ± 2.01 × 10^−2^	3.13 × 10^−2^ ± 2.02 × 10^−2^	0.01 ± 9.00 × 10^−3^	<0.002
Lead ***	mg/L	0.02	<0.02	<0.02	<0.02	<0.02
Mercury ***	mg/L	2.00 × 10^−5^	<2.00 × 10^−5^	<2.00 × 10^−5^	<2.00 × 10^−5^	<2.00 × 10^−5^
Molybdenum ***	mg/L	0.01	<0.01	<0.01	<0.01	<0.01
Nickel ***	mg/L	0.01	<0.01	<0.01	<0.01	<0.01
Selenium ***	mg/L	0.005	<0.005	<0.005	<0.005	<0.005
Potassium ***	mg/L	0.1	7.09 ± 0.202	20.8 ± 21.7	7.01 ± 0.501	9.30 ± 2.14
Zinc ***	mg/L	0.005	7.06 × 10^−2^ ± 3.17 × 10^−2^	0.107 ± 3.31 × 10^−2^	2.23 × 10^−2^ ± 7.32 × 10^−3^	2.89 × 10^−2^ ± 4.59 × 10^−3^

NM = data were not measured; ** Total Suspended Solids (TSS) was measured in lab on the samples collected for analysis; *** Samples were measured only every 3–4 months, otherwise samples were measured biweekly throughout the year.

## Supplementary Materials

The following are available online at https://www.mdpi.com/2076-2607/7/12/626/s1, Figure S1: Map of the Service Areas of the two WWTPs, biological aerated filter (BAF; right) and in Kingston, Ontario with land use designation., Table S1: Summary of Land Use information for the Service Areas of the wastewater treatment plants., Table S2: Gene targets, primers and probes, amplicon size and standards used for quantitative PCR with references for each assay., Table S3: List of antimicrobials, disk suppliers, antimicrobial content of the disks and the breakpoints to define resistance in this study., Figure S2: Principal component analysis of the primary effluent (A) Graph of sample distribution along the first three principle components that account for 97.5% of the variance; (B) Correlations with PC1; (C) Correlations with PC2; (D) Scree Plot., Figure S3: Principal component analysis of the final effluent. (A) Graph of sample distribution along the first three principle components that account for 65.1% of the variance; (B) Correlations with PC1; (C) Correlations with PC2; (D) Scree Plot., Table S4: Number of antimicrobial agents to which enterococci isolates from the primary and final effluents from biological aerated filter and the conventional activated sludge wastewater treatment plants exhibit resistance. Table S5: Binary antimicrobial resistance (AMR) profiles from the 308 enterococcal isolates in this study organized by sample type (primary (PE) and final effluents (FE) of the biological aerated filter (BAF) and the conventional activated sludge (CAS) system and biomass for both WWTPs). The AMR profile is described based on resistance (R), intermediate resistance (I), and susceptibility (S) to 12 antimicrobials including vancomycin (VNCO), teicoplanin (TEIC), ampicillin (AMPI), doxycycline (DOXY), erythromycin (ERTH), nitrofurantoin (NTRO), gentamicin (GENT), linezolid (LNZD), levofloxacin (LVFL), quinupristin/dalfopristin (QUIN) streptomycin (STEP) and tigecycline (TGC). The green fill indicates that that profile is found in all the primary and final effluent samples. The blue fill indicates that the profile is one found in isolates from the BAF system. The orange fill indicates that the profile is only detected in isolates from the primary effluents and the red fill indicates that the profile is only detected in isolates from the final effluents. The fill of other colors indicates that that profile is only detected in one sample type.
